# The Impact of Ethnicity on Objectively Measured Physical Activity in Children

**DOI:** 10.1155/2013/757431

**Published:** 2013-01-22

**Authors:** Emma Lisa Jane Eyre, Michael J. Duncan

**Affiliations:** Department of Biomolecular and Sport Sciences, Coventry University, James Starley Building, Priory Street, Coventry CV1 5FB, UK

## Abstract

Obesity and obesity-related diseases (cardiovascular disease/metabolic risk factors) are experienced differently in individuals from different ethnic backgrounds, which originate in childhood. Physical activity is a modifiable risk factor for obesity and related diseases. Both physical activity and metabolic risk factors track to adulthood, and thus understanding the physical activity patterns in children from different ethnic backgrounds is important. Given the limitations of self-report measures in children, this study provides a review of studies which have objectively measured physical activity patterns in children from different ethnic backgrounds. From a total of 16 studies, it can be concluded that physical activity does seem to vary amongst the ethnic groups especially South Asian and Black compared to White EU (European Union). The findings are less consistent for Hispanic/Mexican American children. However, there are several methodological limitations which need to be considered in future studies. Firstly, there is a need for consistency in the measurement of physical activity. Secondly, there are a range of complex factors such as socioeconomic status and body composition which affect both physical activity and ethnicity. Studies have failed to account for these differences limiting the ability to generalise that ethnicity is an independent risk factor for physical activity.

## 1. Background

Physical inactivity is the fourth leading risk factor for global mortality [1]. It is well understood that engaging in regular physical activity (PA) has beneficial physiological and psychological effects on health and well being in adults and children [2–5]. Specifically, the protective mechanism of PA for the primary and secondary prevention of obesity-related diseases including cardiovascular disease [6, 7] and type 2 diabetes mellitus (T2DM) [2, 8] is well established. This is because PA results in numerous physiological responses which improve cardiovascular fitness and health in adults [1, 7] and children [9, 10], healthy and those with cardiovascular disease [11]. 

Large observational studies show that physical inactivity is related to the progression to T2DM from normal glucose tolerance [12–14]. Engaging in regular PA may be able to delay the progression from impaired glucose tolerance to T2DM [15]. A systematic review and meta-analysis of randomised control trials concluded that exercise (150 mins/week) improved diabetes control with a reduction in HBA1c concentrations in adults with T2DM [16]. Daily walking alone has been associated with improvements in insulin sensitivity and glucose tolerance in adults [17]. 

 Several recent reviews have also concluded that increasing PA can reduce metabolic risk factors in children. Andersen et al. [18] concluded that PA results in a lowering of blood pressure and healthy lipid profiles in children. A meta-analysis of 14 studies found that accelerometer-measured PA was associated with cardiometabolic risk factors [19]. Thus, children engaging in more moderate and vigorous physical activity (MVPA) have better cardiometabolic risk factors [19, 20]. Gutin and Owens [20] concluded that PA interventions in obese children (150–180 mins/week MVPA) were associated with favourable biomarker change. A review by Tompkins et al. [21] supports the role of PA in reducing the incidence of T2DM without weight loss. PA is thus described as an independent risk factor for metabolic syndrome in children independent of other factors [22]. 

Metabolic disease and risk factors for metabolic disease vary by ethnic group and show higher prevalence in non-European groups [23]. Compared with White EU, people from African Caribbean backgrounds evidence lower cardiovascular disease mortality rates but experience higher risk of hypertension and T2DM [24–28]. British South Asian (SA) adults when compared to White EU also experience an increased risk of T2DM which is associated with increasing cardiovascular disease death rates [29–32]. 

In the last 10 years, growing evidence has shown that early markers of metabolic disease risk emerge in childhood which are strongly related to ethnicity and obesity [33–36]. Ethnic differences have been found in body composition between White EU compared to Black [37, 38] and SA [39, 40]. Research also reports that SA and Black children have higher levels of fasting insulin than those of White EU children [41]. Ethnic differences in metabolic risk factors between Black, Hispanic, and White were also reported by Casazza et al. [42]. This is important because Camhi and Katzmarzyk's [43] review has shown tracking of risk factors track from childhood into adulthood.

In adults, studies in the UK and USA have shown that PA patterns are different amongst ethnic groups but highest in White adults [44, 45]. Fischbacher et al.'s [46] review of 12 studies suggests that SA adults are less active, with the majority of this information deriving from self-report measures. Self report in young children is not valid or reliable because children lack the ability to accurately recall PA patterns [47–49]. Questionnaires may also be interpreted differently across cultures. A review in the measurement of PA in children using indirect and direct measures of PA evidenced that there are large discrepancies in children [50]. Further reviews in the measurement of PA consistently report that objective assessment in children is the most accurate and reliable source of information [51–56]. 

PA patterns in adults have also been directly compared to metabolic risk. A study found that Black adults in the highest quartile for PA had 34% lower risk of developing hypertension over 6 years. Pitanga et al.'s [57] review also suggests that 185 minutes of PA for Black men and 215 minutes for Black women were the best cut-points for predicting the absence of diabetes. Williams et al.'s [32] study in SA adults showed that 21% difference in cardiovascular disease could be explained by PA. 

Ethnic differences in adult metabolic disease and PA patterns are apparent. It is also known that PA can explain large proportions of ethnic differences in metabolic disease. Given that the development of metabolic diseases emerges in childhood and tracks to adulthood, it is imperative that a review is conducted to understand ethnic differences in childhood PA. Some studies have considered PA in ethnic groups, but there are differences in the measuring instruments of PA and interpretation of the data, and so no clear consensus on PA between ethnic groups is known. To provide the most accurate and reliable information on PA in ethnic groups of children, this paper will consider only objectively measured PA between ethnic groups of children. Knowing PA differences between children from different ethnic groups could inform ethnic-targeted interventions to increase PA and reduce ill health associated with lack of PA in ethnic groups. 

The purpose of this paper was to examine objectively measured PA in Black, Mexican American/Hispanic, and SA children and compare them to White EU to establish whether there are differences in physical activity patterns based on ethnic groups to inform physical activity interventions which will improve metabolic health.

## 2. Method

An electronic search was conducted from January 1990 until July 2012 using Pubmed, Embase, and Cochrane Database of Systematic Reviews. A child was defined as a subject who was under the age of 18 years in accordance with the UN convention on the right of children (UNICEF, 1989). The following search terms were used: PA, inactivity, sedentary time, exercise, ethnicity, race, ethnic minority, racial, race, ethnic, ethnicity, and children. Searches were limited to those published in the English language.

### 2.1. Selection

**Figure 1:**
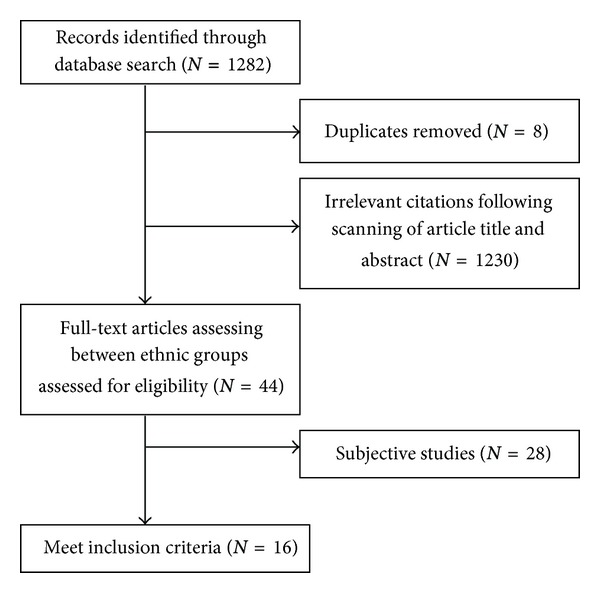
Flow chart of selection process.

Studies were included if PA was assessed objectively and if these findings were reported separately for ethnic groups. Forty-four studies compared PA between ethnic groups: of these, 28 measured physical activity subjectively and thus were removed (Figure 1). Full-text articles were obtained for a total of 16 studies, and the following data were extracted from each paper: study design, country of origin, sampling method, instrument for gathering ethnic information, population characteristics, measuring instrument, outcome measures, and results (Table 1).

### 2.2. Ethnic Criteria

For the purpose of this paper, children are referred to as Black which includes Black, African American, non-Hispanic Black, Black African, and African Caribbean. White ethnic origin was used to describe children from Non-Hispanic White, White European, Caucasian, European American, Anglo Saxon, or White backgrounds. SA ethnicity group included children with ancestral origins in India, Pakistan, or Bangladesh. Finally, Mexican American/Hispanic was defined as Hispanic, Mexican American, Latino, and Hispanic American descent. 

## 3. Results

A total of 16 studies were included, 13 studies were from America, two studies from the UK, and one from New Zealand (Table 1). The sample sizes varied widely from 169 to 3381 participants. Seven of the studies included a sample population above 1000 subjects. The studies reported using mainly a cross-sectional design. Results are presented under ethnic grouping. 

### 3.1. Study Information

A total of 13 studies assessed PA in Black children compared to White EU. Twelve of these were conducted in the USA with only one conducted in the UK [66]. Seven of these studies recruited from multicentres [62, 66–71]. A total of 9 studies included participants from Mexican American/Hispanic descent [42, 63, 67–71, 73, 74]. All these studies were conducted in the United States. Of these, six studies included multicentres of recruitment [62, 67–71].

Despite SAs experiencing the highest risk of metabolic disease, there were few studies examining PA patterns in this ethnic group. Two studied a mix of Indian, Pakistani, or Bangladeshi [65, 66], and the other study included predominantly Indian (91.5%), Sri Lankan, Bangladeshi, and Nepalese [75]. These studies combined ethnic subgroups. Owen et al. [66] considered the effect of ethnic subgroups but did not report statistical information for this. 

### 3.2. Age and Gender

For Black, three studies used girls only [62, 69, 71], and the remaining studies used mixed groups of boys and girls. Two studies assessed PA in preschool children (3–5 years) [58, 72], Five studies included children aged 11 years or younger [59, 66, 68, 70, 73], and two studies included young people aged 12 or more [62, 69]. The remaining studies used a wide age range of participants including both pubertal and prepubertal subjects [42, 67, 71, 74]. 

For Mexican American/Hispanic, studies recruited participants from 6–18 years: of these studies, two examined prepubertal children [63, 68], two examined children aged 12 years or above [69, 71], and five reported combined aged groups [42, 67, 70, 73, 74]. Two out of the ten studies used girls only [69, 71]. Studies in SA children used mixed samples of boys and girls. Two studies focused on SA children 9-10 years [65, 66], and the remaining study included SA children aged from 6 to 16 years [75]. 

### 3.3. Collection of Ethnic Information

In Black children, six studies gathered ethnic information using parent report or self-report [42, 62, 66, 69, 72, 74]. Three reported their criteria for defining ethnic grouping [42, 66, 69]. The remaining studies failed to describe how they gathered information on ethnic background. In Mexican American, five studies reported the method of gathering ethnic information and reported their criteria for ethnic reporting. This was either parental or self-reported [42, 63, 67, 71, 74]. 

### 3.4. The Measurement of PA

PA was assessed in 16 studies of which eleven used Actigraph accelerometers and described the cut-points used to determine time spent in activity such as sedentary or moderate and vigorous [42, 59, 66, 67, 69–74]. The most commonly reported threshold criteria related to Trost et al. [60] or Treuth et al. [61] validation studies. One study used a Caltrac accelerometer [62]. PA was assessed by a pedometer in three studies [65, 68, 75] and observational analysis in one [58].

### 3.5. PA: Activity Counts/Steps

Considering pedometer determined PA, one study reported that Black children were less active, engaging in fewer recorded steps than White children [68]. However, studies using Actigraph accelerometers evidenced that Black children recorded more counts per minute. This was apparent in prepubertal children [59, 66, 73] and for children ranging from prepubertal to pubertal (6–18 years old) [67]. Newton et al.'s study [59] was the only one of these studies to take into account differences in socioeconomic status (SES) evidencing that low socioeconomic Black girls engage in more counts/min. However, White and Jago [62] found that Black girls had less counts per day than White girls. 

There were two studies which examined PA in Mexican American/Hispanic as counts per minute or total activity using Actigraphs [63, 74], and one study assessed PA using a pedometer as steps per day [68]. These studies were all in children under 12 years. Johnson et al.'s [68] study examined three days of weekday pedometer data and found that Mexican American/Hispanic children aged from 10 to 11 years accumulated less steps per day than White children. They also reported that region affected accumulated steps: children from urban areas accumulated fewer steps than their rural or suburban peers. However, this study did not match ethnic groups from each built environmental area to compare ethnic differences, and this may have affected the outcomes. 

Two smaller studies (147 to 169 children) [63, 74] and one larger study [73] assessed PA using the Actigraph accelerometer. All studies showed no ethnic differences in total PA. Byrd-Williams et al.'s [63] study of children (9.4 years study) reported that 24.5% were overweight and 15.4% obese. However, the effects of weight status were not controlled for during analysis of total PA between ethnic groups. Casazza et al. [42] also reported no difference in total PA or sedentary PA in 7- to 12-year-old children. This study also reported that Mexican American/Hispanic children had higher BMI, increased body adiposity, and lower socioeconomic status than White peers. Again, these variables were not controlled for during the analysis. Given the influence of other variables in the previous studies that affect activity but were not controlled for, it is not possible to draw conclusions on Hispanic children's PA patterns assessed as activity counts or steps. 

All the studies that measured PA in SA children found that they had a lower PA count or achieved fewer steps per day [65, 66, 75]. In addition, Owen et al. [66] considered within-ethnic differences in the SA sample, and although SAs did have lower levels of PA as a whole, there were no differences between Indians, Pakistanis, and Bangladeshis. Duncan et al. [65] controlled for BMI, hours of daylight, and age and still reported ethnic differences. They also reported that socioeconomic status affected total activity steps and that this relationship was different on weekdays and weekends. However, ethnicity and socioeconomic status were not considered together. Duncan et al. [75] also reported significant differences in steps with SES as well as age but failed to account for these differences. Duncan et al. [65] was the only study that considered body composition differences and accounted for them. 

### 3.6. PA: Time Spent in PA

#### 3.6.1. Sedentary Time

A large multicentre study considered sedentary time in 6–11-year-old boys and girls and found that Black girls spent fewer hours a day in sedentary activity than White [70]. However, two fairly small studies in prepubertal boys and girls found no significant difference in time spent in sedentary activity [42, 59]. Two large multicentre studies found that Black children were more sedentary than White. Belcher et al. [67] reported that Black children aged from 6 to 11, 12 to 15, and 16 to 19 were more sedentary than White. Owen et al. [66] similarly reported, in children aged from 9 to 10, that Black children were more sedentary. 

Studies used an activity count of less than 100 to determine sedentary time and thus are comparable based on activity. The results are equivocal, and this may relate to some differences in methodology. However, the large multicentre studies considering boys' and girls' sedentary behaviour together suggest that Black children are more sedentary than White children across age groups. However, Kelly et al. [69] reported that White girls, but not boys, were more sedentary. To understand sedentary behaviour in Black and White children more accurately, future studies may need to consider age, gender, and ethnicity. 

There were inconsistent findings regarding sedentary time in Mexican American/Hispanic. Firstly, Casazza et al. [42] found no difference in total daily minutes of PA versus sedentary activity between Mexican American/Hispanic and Caucasian groups. Matthews et al. [70] reported that time spent in sedentary activity (defined as <100 counts min) was lower in Mexican American/Hispanic groups than in Caucasians. This study used the Treuth et al. [61] equation in adolescents to determine sedentary behaviour. Belcher et al.'s [67] secondary analysis of National Health and Nutritional Examination (NHANES) data accounted for age, unlike Matthews et al. [70], and suggested that Mexican Americans/Hispanics were more sedentary than Whites. Owen et al. [66] was the only study which considered sedentary PA and reported that SA children are more sedentary than White children. 

#### 3.6.2. Time Spent in Light PA

Information regarding Black and SA children time spent in light PA was not assessed in any studies. Byrd-Williams et al. [63] were the only authors who considered light PA, finding no difference in mean percentage of monitored time spent in light PA in Mexican American/Hispanic. 

#### 3.6.3. Time Spent in MVPA

For time spent in MVPA in Black and Mexican American/Hispanic girls, the findings were conflicting. A large multicentre study in girls (aged 12 years) found that Black and White girls spent similar amounts of time in MVPA [69]. The study also reported no difference in time spent in MVPA between Mexican American/Hispanic and White girls [69]. Another multicentre study reported that Black girls and Mexican American/Hispanic girls spend less time in MVPA than White (10–13 years) [71]. The differences may thus be explained by a number of reasons, such as different population characteristics in relation to age and ratio of White to Black children and the inclusion criteria for activity monitoring. 

In addition, Kelly et al. [69] reported that Mexican American/Hispanic had higher BMI than White, but failed to control for this during analysis. Pate et al. [71] did not report BMI or any other measure of body composition. This study also considered three different cut-points and different sampling periods (30 and 60 minutes MVPA) and suggested that ethnic differences were dependent upon the cut-points applied. No significant ethnic difference was found for 60 minutes MVPA describes using ≥4.6 METS, ≥3.8 METS, and ≥3.0 METS. However, ethnic differences were found for 30 minutes MVPA for ≥4.6 METS and ≥3.8 METS. The statistics were not displayed separately for White and Mexican American, but the means show that higher number of White children achieved these guidelines. Fewer children engage in large amounts of daily activity which may explain the lack of differences at 60 minutes. The study highlights the importance of accelerometer cut-points, but to date no broad consensus has been met on what to use. 

For Black boys and girls combined, the findings were more consistent. One small study suggested that Black and White children engage in similar MVPA [42]. However, the remaining five studies found that Black children spend more minutes in MVPA [59, 66, 67, 72, 73]. This was found across a range of ages including preschool [72], prepubertal [59, 66, 73], and prepubertal and pubertal [67]. Of these five studies, four used large multicentre sampling. All five studies used Actigraph accelerometers to assess PA but employed different thresholds to determine MVPA. The results from larger studies of both boys and girls would, therefore, suggest that Black children spent more time in MVPA. 

The findings in Mexican American/Hispanic boys and girls combined were less consistent. Two studies reported that Mexican American/Hispanic children engage in fewer minutes of MVPA than White EU [42, 74]. However, two studies also reported no difference in mean percentage of time in MVPA [63, 73]. Again, these studies used different accelerometer thresholds for MVPA. Casazza et al. [42] used the software calculations to report time spent in different intensities but did not report how these are calculated with reference to MET levels or activity counts that translate to MVPA. Casazza et al. [74] define MVPA as anything greater than 3 METS. Byrd-Williams et al. [63] used the Puyau et al. [64] accelerometer cut-points which defines MVPA as counts greater than or equal to 3000 or greater than/equal to 0.5 kcal/kg/min (equivalent to 3 METS or more), developed from data from 6–16-year-old children. 

However, Byrd-Williams et al. [63] firstly accounts for age by focusing on 9-10 years unlike the other two studies which include children aged from 7 to 12 years. They report no differences in sex, height, weight, or age. Yet, almost half of the samples (43%) were overweight or obese. Body composition differences between ethnic groups were also reported [42, 74], but none of the studies controlled for these. All of the three studies also had uneven samples of Mexican American/Hispanic and White, with more White people taking part, and were based on small samples (<250 in total). The small sample sizes also limited the analysis in a number of ways. Furthermore, Byrd-Williams et al. [63] report gender differences in MVPA, but the sample size restricted exploring for gender and ethnicity interaction effects as well as controlling for differences in BMI. Secondly, SES differences were reported [42, 74], whereby Mexican American/Hispanic children had lower SES status, but again the small sample size limited the ability to assess these effects within ethnicity on PA counts, and thus it is not possible to state that ethnic differences are independent of other influences. 

Large studies have shown that Mexican American/Hispanic children spend more minutes in MVPA [67]. Belcher et al. [67], using data from NHANES, also reported results for boys and girls separately but found no significant difference in Hispanic/Mexican American girls' versus White girls' engagement in moderate to vigorous activity but found that Mexican American/Hispanic boys spent more time in MVPA. They reported results separately for age groups as well suggesting that differences in activity patterns between Hispanic/Mexican American and White exist in 6–11- and 12–15-year-old children but that there were no differences found in children aged 16–19 years. The study also accounts for difference in weight status with ethnic groups PA. The only ethnic differences found were for overweight children aged 16–19 in which White were less active than Hispanic. Belcher et al. [67] used accelerometer thresholds by Trost et al. [60] which provide thresholds based on age. Their study highlights that ethnic differences in activity patterns are likely to be dependent on age, gender, and adiposity. Belcher et al. [67] also reported a three-way interaction between age, BMI, and ethnicity for minutes in MVPA, evidencing that overweight Hispanic/Mexican American boys and girls spent less minutes in MVPA than non-overweight. This relationship might be complex and might explain some of the inconsistencies in the findings, which makes it difficult to draw conclusions from the data. 

For SA children, the findings consistently reported that they were less active than White children. Duncan et al. [65] used a pedometer and cut-points of 15,000 for boys and 12,000 for girls to determine whether children met PA guidelines (60 mins/day). Their study found that a lower proportion of SA children met the guidelines for health. Owen et al. [66] also reported that lower numbers of SA children met the guidelines. They also found that SA children spent less time in MVPA. The findings in SA children would suggest that SA children are less active than White. 

## 4. Discussion 

There are few studies that have examined ethnicity and objective PA in children. The majority of research is based on cross-sectional studies predominantly from the USA. There is limited information about PA patterns from the UK and few studies considering SA ethnic children. 

The results would suggest that Black children are more active based on activity counts and time spent in moderate and vigorous PA [59, 66, 67, 73] but that they also spend more time in sedentary activity [66, 67]. These findings, however, might be different for Black girls, and more high quality studies are needed to understand gender, age, and ethnicity effects on PA especially sedentary activity in Black children. 

For Hispanic/Mexican American children compared to Whites, the results are equivocal. This may be explained by methodological limitations and failure to account for influential factors such as age, gender, body composition, and SES which made it impossible to conclude whether there were genuine differences in PA between Hispanics and Whites. More high quality studies are needed in Hispanic/Mexican American populations which control for confounding factors in order to gain a clearer understanding of PA patterns. 

There were fewer studies in SA children, but the studies that were available consistently reported that SA children were less active engaging in fewer minutes of MVPA, more sedentary time, and being less likely to meet PA guidelines [65, 66, 75]. 

### 4.1. Methodological Limitations

There were a number of methodological differences among the studies which made it difficult both to analyse the results and also to be able to compare between study findings. 

#### 4.1.1. The Measurement of PA

The measurement of PA is a complex phenomenon, especially in children. Firstly, the objective measurement of PA is a strength of the review and in particular the studies used accelerometer data which is viewed as an accurate way to measure PA in children. Accelerometers make it possible to measure PA count as well as intensity and frequency of PA whilst eliminating human error. However, accelerometers do not capture all types of PA especially nonweight bearing activities like swimming or cycling [60]. When examining the intensity of PA, it is possible to assess whether children are fulfilling national/international guidelines for levels of PA which are described in terms of MVPA. 

There are also differences in the way PA has been reported. Pedometers consider total activity steps, but accelerometers consider mean activity counts per minute. For accelerometer data specifically, these mean activity counts daily are then converted to time spent in sedentary activity, light activity, or MVPA using accelerometer cut-points. However, not all studies report each of these elements. The accuracy of some of the conversions needs to be considered because different accelerometer cut-points are applied which have been validated on a wide range of children of differing age and gender, which has not been considered. There are also no studies that consider ethnicity in the validation of these thresholds. Currently, there is no standardised measure of accelerometer cut-points which makes it difficult to compare between PA studies. This has made it difficult to determine whether PA differences do exist or whether differences are due to differences in the criteria used in each study. A recent systematic review of objective measures of PA measurement reanalysed all PA data in children using the three most popular cut-points [54]. They found significant differences in the amount of sedentary behaviour and MVPA when different cut-points were used suggesting that the assessment of engagement in PA is dependent on the cut-points applied to the data [54]. 

The studies also varied in the measuring time for periods of activity. Studies used mainly 60, 30, or 15 second intervals. The nature of children's PA is intermittent, and thus using longer epochs may fail to describe the true nature of these patterns. Reilly et al.'s [54] systematic review also reanalysed all PA data in children using 15, 30, 45, and 60 second intervals. They found that there was a small but significant difference in the results using different intervals but concluded that there was still limited evidence concerning the use of long or short measuring periods. The study made recommendations that moderate and vigorous PA should be analysed together in order to reduce misclassification from epoch lengths. This paper considers moderate and vigorous PA together to minimise the effect of choice of epoch on PA.

The studies also varied in the number of days used for monitoring ranging from 1 day to 7 days. The period of data collection that has been included has also varied from 5 hours to less than 18 hours. This has important implications as data reliability is reported to increase with an increased number of days of monitoring. Seven days monitoring with inclusion criteria of 10 hours per day of data is reported to be the most reliable [76]. Out of the 13 studies, five reported inclusion criteria of 10 or more hours [59, 66, 67, 70, 71]. The remaining studies inclusion criteria were less than 10 hours or failed to report them. This may affect the validity and reliability of the results produced. Future studies should include a minimum of 10 hours. 

It is also important for studies to gain a whole picture when examining PA patterns. For example, studies in Black children found that Black children were more sedentary. This would suggest that they are less active. However, studies have also reported that Black children were more active. Thus by considering only one component of PA, it can cause overgeneralisation about PA patterns which might not be a true indication of activity patterns. By gaining the whole picture, it is possible to conclude that Black children can be both more active and more sedentary, and thus their activity patterns are likely to lie at both extremes. In addition, the majority of studies have unequal, usually smaller numbers of ethnic groups, and do not report response rates in ethnic groups. It is not known whether these are representative samples of ethnic groups, which limits the ability to generalise the results. 

### 4.2. Age/Gender

The studies consider a range of ages from preschool, prepubertal, and adolescent and in some cases mixtures of age groups. Yet, during analysis, very few studies have accounted for age differences. However, looking at age differences separately, a study has found differences in PA in different age groups of children [67]. Age differences in PA have also been described in other studies [60, 77]. Gender differences in PA patterns have also been described in studies [66, 78]. The majority of studies in this paper sampled both boys and girls, combining groups to compare ethnic differences. A few studies considered girls only, but none considered only boys. From these studies, the interaction effects between ethnicity and gender on PA remain unclear. It is likely that reporting results separately for gender and age groups would yield different results. 

### 4.3. Ethnicity

Not all studies reported how they gathered ethnic information or how they defined ethnic groups. In studies that did report this information, ethnicity was gained from self-report measures. A few studies also defined how ethnicity was defined. There are differences in assessing ethnicity, and there are likely to be differences in tracing ethnicity back more than 3 generations to current generations. In addition, the studies and this paper do not consider ethnic heterogeneity, providing data on ethnicity as a homogenous group. For instance, there are likely to be differences in SA subgroups such as Indian, Pakistani, and Bangladeshi. Only Owen et al. [66] considered this (but did not report the statistical results). This does significantly increase the size of the sample that needs to be examined in any given study. There are also likely differences in “Mexican American born” compared to “immigrant born” Hispanic PA patterns. There may also be differences in White ethnic groups such as those born in the UK, those from the USA or those with another European background. This paper also only makes comparisons between Black, Mexican American/Hispanic, or SA children compared to White children. The difference between other ethnic groups is not known due to paucity of data on these ethnic groups. 

Gaining nationally representative information in ethnic groups is a challenge. Ethnic groups tend to cluster in sociodemographically deprived areas, and it becomes difficult to generalise the data to a wider population. Some studies provided in this paper did consider SES and found differences for ethnic groups with White being less disadvantaged [42, 59, 74, 75]. However, few studies accounted for SES during analysis. Newton et al.'s [59] study suggests a difference in PA between low SES Black and low SES White but no other differences relating to SES, ethnicity, and PA. Duncan et al. [65] reported differences in SES status on weekdays and weekends, suggesting that high SES engaged in less PA on weekends but more on weekdays. Given that ethnic groups tend to cluster in the most disadvantaged areas, SES is an important covariable. A study found that poverty was increased in non-White neighbourhoods [79]. Additional studies have reported that SAs are the most socioeconomically deprived and are likely to live in the most deprived areas [80]. A number of studies have also associated deprivation with physical activity [81–87]. The reasons for differences in patterns of PA may thus be more complex with environmental factors being a determinant for PA. For instance, the environment may affect the opportunities available to be physically active but this may depend on SES. Perceptions about safety, facilities, and spaces and neighbourhood walkability are just a few environmental factors associated with physical activity [88–92]. However, there appear to be no studies examining environmental influences that have considered ethnicity, and thus it is hard to ascertain whether PA differences by ethnic groups are representative of environmental and SES differences rather than ethnic differences per se. 

Finally, the role of body composition on PA in ethnic groups needs to be considered. Ethnic differences in body composition have been reported [35, 37, 62, 93–96] as have the differences in PA with body composition [42]. In this paper, body composition was assessed by proxy using BMI which can be problematic in ethnic groups that have different amounts of fitness recommendations. 

This paper highlights a number of important recommendations. Firstly, studies need consistency in methodology especially the need for universal accelerometer cut-points thresholds. Secondly, future studies would benefit from controlling for age, gender, SES, and body composition. Thirdly, there is a need for UK-based studies as the effect of ethnicity might be different depending on the country. Finally, future studies should also consider longitudinal and experimental designs to enable more inferences about PA and the development of metabolic disease. Understanding ethnic differences in PA will enable PA interventions to be focused at specific ethnic groups to increase PA. 

## 5. Conclusion

This paper does suggest that there are ethnic differences in PA for Black and SA children, but findings are unclear for Mexican American/Hispanic. It is not clear whether these differences are due to physiological, cultural, or environmental differences or a combination of these factors. Future studies with improved methodology are necessary to examine the impact of low levels of PA on cardiometabolic risk factors in childhood and to develop effective ethnically sensitive interventions to promote physical activity in risk groups. 

## Figures and Tables

**Table 1:** Studies objectively assessing PA between ethnic groups.

Author	Total number, age, and ethnic group	Instrument	Thresholds	Outcome measures	Results
Black					

Pate et al. [58] USA	Total = 438 3–5 (4.2 ± 0.7) Boys (50%) and girls (50%) Black 58.7% White 41.3%	Observation analysis	N/A	Frequency of each activity rated on scale 1 to 5. 30 min sessions, 10–12 sessions = 600–720 session per child	**Light/sedentary:** No significant ethnic difference (adjusted for BMI). **MVPA:** No significant ethnic differences in percentage of time MVPA, and active (*P* > 0.05) adjusted for BMI.
Newton et al. [59] USA	Total = 272 Mean age = 10.4 ± 1.1 African American (207), 66 boys and 141 girls Caucasians (65), 26 boys and 39 girls	Actigraph	Trost et al. (2002) [60] Treuth et al. (2004) [61] for sedentary PA	Activity counts per minute Time spent in MVPA, light, and sedentary activity **Inclusion:** 2 days, 10 hours, 30 sec epoch, weekdays only	**PA:** Low SES Black engaged in more counts per minute than White (*P* < 0.05). **Light/sedentary time:** No significant difference between Black and White. **MVPA:** Low SES Black spent more time in MVPA than low SES White (*P* < 0.05).
White and Jago [62] USA Multicentre	Total = 1148 12 years Black 538 White 610 girls	Caltrac		Activity counts per day Inclusion: 2-3 days	**PA:** Black girls achieved less counts per day than White girls (*P* < 0.001).

Mexican American/Hispanic					

Byrd-Williams et al. [63] USA	Total = 169 Mean age 9.4 years Hispanic (63%) Non-Hispanic White (37%)	Actigraph	Puyau et al. (2002) [64]	Activity counts per minute Hours spent in MET levels **Inclusion:** 2 or more days, more than 6 hours, 1 min, wake hours	**PA:** No difference in total physical activity (*P* = 0.08). **Light/Sedentary:** No difference in light of sedentary physical activity (*P* > 0.05). **MVPA:** No difference in time spent in MVPA (30 mins), light, or sedentary PA (*P* > 0.05).

South Asian					

Duncan et al. [65] UK	Total = 563 9.6 ± 1.0 White South Asian 255 boys and 281 girls	Pedometer	15,000 boys 12,000 girls	4 days	**PA:** White engaged significantly in more steps/day than SA. **MVPA:** A lower proportion of SA met the 60 minutes of MVPA guideline.

Mixed Ethnic Groups					

Casazza et al. [42] USA	Total = 202 Age 7–12 years) White 68 African American 79 Hispanics 55	Actigraph Body composition-DXA	Actigraph software	Minutes in MVPA, vigorous, sedentary, and moderate activity **Inclusion:** 1 minute epochs, wake hours (7 days)	**Total mins PA/sedentary:** No significant difference in daily minutes of total activity or sedentary activity between ethnic groups (total PA = White 670.88 ± 16.4, Black 670.94 ± 16.9, Mexican American/Hispanic 661.08 ± 18.8 (min/d), sedentary = white 438.07 ± 9.2, Black 423.97 ± 10.4, and Mexican American/Hispanic 431.53 ± 10.8 (min/d)) (*P* > 0.05). **MVPA:** White spent more minutes than Hispanic (MVPA = White 69.22 ± 4.5, African 59.87 ± 4.4, and Hispanic 51.07 ± 4.9 (min/d)) (*P* > 0.05). No difference between Black and White.
Owen et al. [66] UK	Total = 2144 Age 9-10 years White EU 565 SA 494 Black African 607 Others 408	Actigraph	Mean daily times (minutes) spent in sedentary (defined as <100 CPM), light (100 to <2000 CPM), moderate (2000 to <4000 CPM), vigorous (44000 CPM), and moderate to vigorous physical activity (MVPA)	Mean counts per minutes, daily steps Time spent in MET levels Mean registered time Measured: 30 sec **Inclusion:** >600 minutes data collection	**PA:** South Asians recorded fewer counts per minute and daily steps compared to White. Black recorded significantly more counts per minute than White. **Sedentary time/light:** South Asians and Black children spent more time in sedentary than White. Black spent more time in light activities. **MVPA:** South Asian children recorded fewer minutes in moderate and vigorous levels of physical activity (*P* = 0.02). Black children spent more time MVPA and vigorous PA (*P* < 0.0001) and recorded fewer steps compared.
					*60 mins day MVPA (government recommendations):* lower proportion of South Asians achieved 60 minutes of MVPA (54%) compared to Whites (70%) and Blacks (69%), but gender differences were not modified by ethnicity (*P* > 0.07). Black and White showed no difference.
Belcher et al. [67] USA **NHANES** **03-04** **05-06** Multicentre	Total = 3106 (boys 1508/girls 1598) (Age 6–19 years) Non-Hispanic Black 605/522 Non-Hispanic White 416/428 Mexican American 548/1587	Actigraph	Trost et al. (2002) [60]	Mean counts per minute Minutes spent in MVPA Inclusion: 4 days, 10 hours minimum per day of data collection	**P** **A** *Overall:* Whites had lower mean counts than Blacks and Mexican American/Hispanics (*P* = 0.05). Overweight Black children had higher counts per minute than Whites (*P* = 0.002). Black children aged 16–19 years were the most active. Mexican American/Hispanic boys spent more time in MVPA, and vigorous PA than White boys (*P* < 0.05). No differences between ethnic groups and girls sedentary, MVPA and vigorous activity. *6–11 years:* Black children and Mexican American/Hispanic were more sedentary than White (*P* < 0.05). No difference between Whites and Mexican American/Hispanics in moderate, vigorous, or MVPA. Black spent more time in moderate activity, vigorous activity, and MVPA than Whites (*P* = 0.05). *12–15 years:* Whites were less sedentary than Blacks and Mexican American/Hispanics (*P* = 0.05). Blacks spent more minutes in moderate activity, vigorous activity, and MVPA than Whites. Mexican American/Hispanics spent more minutes in moderate, vigorous, and MVPA than Whites. *16–19 years:* Whites were less sedentary than Blacks. No difference between Whites and Mexican American/Hispanics. Blacks spent more time in moderate activity than Whites.
Johnson et al. [68] USA Secondary analysis multicentre	Total = 582 Mean age 10.37 ± 0.48 years Hispanic 307 Caucasian 150 African American 125 Urban, rural, and suburb	Pedometer		Steps per day **Inclusion:** 3 days	**PA:** White girls averaged more steps per day than Mexican American/Hispanic and Black (*P* < 0.05). No significant differences between boys.
Kelly et al. [69] USA Multicentre	Girls only Total = 1180 Age 12 years Black 24.5% Hispanic 15.7% White 59.8%	Actigraph	Treuth et al. (2004) [61]	MVPA time (30 sec) 6 days	**MVPA:** No significant difference in Mexican American/Hispanic girls and Black girls MVPA compared to White girls (Mexican American/Hispanic = 21.7 minutes per day, Black 19.5 minutes per day, and White 22.8 minutes per day, *P* = 0.100).
Matthews et al. [70] USA NHANES 03-04 multicentre	Total = 1612 *6–11 years boys 360 (girls 404)* Non-Hispanic Whites 104 (117) Non-Hispanic Blacks 138 (149) Mexican Americans 118 (138) *12–15 years boys 447 (girls 401)* Non-Hispanic Whites 121 (105) Non-Hispanic Blacks 180 (145) Mexican Americans 146 (151)	Actigraph	Treuth et al. (2004) [61]	Amount of time spent in sedentary behaviour (hours/day) **Inclusion:** 7 days, 1 minute epochs, 10 hours of data collection.	**Sedentary activity:** 6–11 yrs: Black girls were less sedentary (5.88 ± 0.06) than White (6.18 ± 0.06) (*P* < 0.05). Mexican American/Hispanic were less sedentary than Whites (*P* < 0.05)
Pate et al. [71] USA Multicentre	Girls only Total = 1578 Mean age 11.7 ± 0.4 to 12.3 ± 0.7 years 706 Whites 344 African American 343 Hispanic 61 Asian American	Actigraph	Treuth et al. (2004) [61] + 2 others	Compliance to 30 minutes, a day recommendation **Inclusion:** 6 days, 30 sec, 12 hours data collection.	**MVPA:** White girls were more active (MVPA) than other ethnic groups (Black and Mexican American/Hispanic, (*P* = 0.01)).
Pfeiffer et al. [72] USA	Total = 331 (Age 3–5) White 40.2% African American 51.4% Others 8.5%	Actigraph	Pate et al. (2006) [71]	MVPA min·hr Nonsedentary activity Measured: 15 second intervals, 5 days **Inclusion:** >5 hours and <18 hours data collection	**PA:** Black children were more active than White children (MVPA = 7.9 versus 7.3 min/hr, *P* < 0.05). Black males were more active than White males (MVPA = 8.5 versus 7.8 min/hr *P* = 0.05).
Gortmaker et al. [73] USA	Total = 3381 1665 and 1716 (6–11 years) Non-Hispanic Black 1232Non-Hispanic White 901 Mexican 1248	Actigraph	Trost et al. (2002) [60]	Means counts/min Mins/day MVPA, 4 days or more. 1 min	**PA:** Non-Hispanic Blacks were more active than non-Hispanic Whites (*P* < 0.01) (accelerometer counts). **MVPA:** Non-Hispanic black more minutes in MVPA than non-Hispanic white spent (adjusted). Hispanic versus White showed no significant difference (adjusted). Time in activity increased in non-Hispanic White but decreased in Black and Mexican children 1 year later.
Casazza et al. [74] USA	Total = 215 Age 7–12 years African American 68 European American 92 Hispanic American 55	MTI Actigraph Body composition- DXA	Moderate (3–5.9) Hard (6–8.99) Hard >9 Light ≤2.99.	Daily counts Minutes in METS Measured: 7 days, 1 minute epoch	**PA:** No difference in total PA. **Light/sedentary:** No difference in sedentary activity between racial/ethnic groups (*P* < 0.05). **Total MVPA:** White children engaged in more moderate and hard activity than Mexican American/Hispanic (*P* < 0.05). **Daily MVPA: ** No difference between White, Black, and Mexican American and Hispanic. No difference in sedentary behaviour. Pubertal status assessed.
Duncan et al. [75] New Zealand	Girls only Total = 1213 (Age 6–16 years) 637 EU 272 Pacific Island 207 East Asian 179 Maori 142 South Asian 76 other ethnic groups	Pedometer (NL-2000)	N/A	Steps per day	No difference in pedometer compliance between ethnic groups. **PA:** South Asian girls were the least active ethnic groups during weekdays and weekend (*P* < 0.005).

MET: metabolic equivalent, MVPA: moderate and vigorous physical activity, DXA: dual X-ray absorptiometry, PA: physical activity, and BHR: basal heart rate.
